# Supplementary material for: Diagnostics to support the eradication of yaws—Development of two target product profiles
Source: PLoS Negl Trop Dis. 2022 Sep 1;16(9):e0010554. doi: 10.1371/journal.pntd.0010554 (PMC9473620; doi:10.1371/journal.pntd.0010554)
Supplement: Attachment — Submitted filename: Responses to reviewer comments.docx [file pntd.0010554.s001.docx]

**Responses to reviewers**


**Reviewer #1:** Minor revision. Figure 1 quality needs to be improved.

Response: We have replaced figure 1. The new figure 1 has a better quality.

Minor revision, WHO reassured criteria should be moved to the discussion.

Response: This has been moved to the discussion section. This now reads in the discussion section as follows:

Ideally, the TPP specifies the need for point of care tests that fulfill the WHO assured criteria. These tests should have a very high sensitivity and specificity (close to 100%) to detect known azithromycin resistance mutations in yaws patients with active infection. To scale up case finding and accelerate effort towards eradication, these tests should also have real-time connectivity, use specimens that are easy to collect, and be affordable, sensitive, specific, user-friendly, rapid, equipment-free and deliverable to those who need it.

We also emphasized this again the conclusion because we consider the statement to be a crucial takehome message.

Minor Comments

1. Neglected Tropical Diseases should be abbreviated in the Abstract.

Response: Done.

2. Line 6 of the abstract: The second "and" should be deleted.

Response: Done.

3. The first and third sentences in the introduction seem contradictory with regard to endemicity of yaws in the tropics

Response: The part of the first sentence that mentions tropics has been deleted.

4. Page 5: Since azithromycin resistance is a major concern, it's important to mention in which geographical location/s resistance has been reported.

Response: We have inserted a sentence about the geographical location. This now reads as:

In Papua New Guinea, long term follow up studies have reported Azithromycin resistant yaws cases 24 months post MDA(Ref 6).

5. Page 5: Suggest changing "one of which" to "the latter"

Response: Done. However, when ‘the latter’ was inserted in the sentence, we do not think the coherence was maintained.

6. Page 6: Methods section, "The seven members" should read "The seven member"

Response: Done

7. Figure 1: The text is illegible.

Response: The entire figure has been replaced with a better quality image.

8. Page 9: Suggest adding POC before lateral flow assays.

Response: Done

9. I suggest moving the statements on WHO reassured criteria to the discussion.

Response: Done. Please see above.

**Reviewer #3**: I recommend the authors add a statement in the discussion indicating that the recommendations will need to be validated in real life situations.

Response: This statement has been added to the discussion and reads:

‘It should be noted that the recommended TPPs have not yet been tested in any of the populations described. As a result, this paper is hypothetical based on a number of well-established assumptions. Therefore, the recommendations will need to be validated in real-life situations.’

Reviewer #3: Fongwen et al, provide a description of the process followed by the working group in coming up with two different target product profile development strategies which will ultimately result in control and eradication of Yaws. The manuscript is well written and the objectives are clear. Whether or not the proposed TPPs are feasible for high risk settings will require extensive field work. The bar which is set for diagnostics in Table 1 and Table 2 is very high and thus may not be achievable in real world situations.

Response: The authors agree with the reviewers. This is a pertinent point. We think the feasibility will be tested in field studies in future studies.
