# Supplementary material for: Diagnostics to support the eradication of yaws—Development of two target product profiles
Source: PLoS Negl Trop Dis. 2022 Sep 1;16(9):e0010554. doi: 10.1371/journal.pntd.0010554 (PMC9473620; doi:10.1371/journal.pntd.0010554)
Supplement: Attachment — Submitted filename: Responses to reviewer comments copy.docx [file pntd.0010554.s002.docx]

**Responses to reviewers**


We have corrected the typos noted by the reviewer
